# Autocrine activity of engineered IL-33 mRNA enhances adoptive T-cell therapy for peritoneal carcinomatosis and synergizes with IL-12 mRNA

**DOI:** 10.7150/thno.122132

**Published:** 2026-01-01

**Authors:** Leire Arrizabalaga, Claudia Augusta Di Trani, Celia Gomar, Daniel Moreno-Luqui, Nuria Ardaiz, Virginia Belsue, José González-Gomariz, David Ruiz-Guillamon, Aline Risson, Yufei Zheng, Eduardo Huarte, Ignacio Matos, Ignacio Melero, Pedro Berraondo, Fernando Aranda

**Affiliations:** 1 Program of Immunology and Immunotherapy, Cima Universidad de Navarra, Cancer Center Clínica Universidad de Navarra (CCUN), Pamplona, Spain.; 2 Navarra Institute for Health Research (IDISNA), Pamplona, Spain.; 3 Centro de Investigación Biomédica en Red de Cáncer (CIBERONC), Madrid, Spain.; 4 Departments of Immunology and Oncology (CCUN), Clínica Universidad de Navarra, Pamplona, Spain.; 5 Nuffield Department of Medicine (NDM), University of Oxford, Oxford, United Kingdom.

**Keywords:** cytokine engineering, immune modulation, T cell therapy, tumor microenvironment, RNA electroporation, solid tumor immunotherapy

## Abstract

**Rationale**: Peritoneal carcinomatosis (PC) remains a major clinical challenge with limited therapeutic options across tumor types. Adoptive cell therapy (ACT) with tumor-specific T cells offers promise, but its efficacy is often impaired by the immunosuppressive tumor microenvironment (TME). Intraperitoneal ACT is under investigation to improve its effectiveness against metastases within the peritoneal cavity. IL-33, a cytokine of the IL-1 family, plays dual roles in immunity and inflammation and may enhance antitumor responses. We evaluated whether IL-33 mRNA-engineered T cells improve ACT efficacy in murine PC models and assessed potential synergy with IL-12 mRNA.

**Methods**: OT.I, PMEL-1, and CEA-specific CAR T cells were electroporated with mRNA encoding IL-33, IL-12, or an IL-33 mutein. *In vitro* assays measured cytokine production and cytotoxicity. RNA-seq was performed to analyze transcriptomic changes following IL-33 mRNA electroporation. ST2^-/-^ T cells were used to evaluate the role of IL-33 receptor expression on transferred T cells versus host cells. *In vivo* studies in murine PC models assessed survival and immune responses using ELISA, ELISpot, and flow cytometry.

**Results**: IL-33 mRNA-electroporated OT.I T cells exhibited enhanced IFN-γ expression in a ST2-dependent, T cell-intrinsic manner. *In vivo*, IL-33-engineered T cells significantly improved survival in PC models. IL-33 reshaped the TME by increasing infiltration of innate lymphoid cells and eosinophils while reducing neutrophils. Engineering T cells with a stabilized IL-33 mutein further enhanced antitumor activity. Co-electroporation of IL-33 mutein and IL-12 mRNA in PMEL-1 T cells led to synergistic increases in IFN-γ production, cytotoxicity, and long-term memory, resulting in superior tumor control and protection upon rechallenge. These findings were confirmed using IL-33 mutein/IL-12 mRNA-electroporated CEA CAR T cells in peritoneal tumor models.

**Conclusions**: IL-33 enhances ACT efficacy by promoting IFN-γ expression via autocrine ST2 signaling and by modulating the TME. The IL-33 mutein improves cytokine stability and antitumor activity, while combination with IL-12 yields synergistic effects. This strategy holds promise for enhancing ACT in peritoneal carcinomatosis.

## Introduction

Peritoneal carcinomatosis (PC) represents the late-stage metastatic dissemination of intra-abdominal malignancies to the peritoneal surface, leading to the widespread presence of tumor cells within the peritoneal cavity. PC is commonly associated with ovarian cancer (75% of cases), colorectal cancer (15%), gastric cancer (14%), and, less frequently, breast and lung cancers (9%) at diagnosis. Additionally, metachronous PC occurs in 20-50% of cases, further complicating disease management 1-4.

The current standard of care for PC includes cytoreductive surgery (CRS) combined with hyperthermic intraperitoneal chemotherapy (HIPEC) in selected cases. While this multimodal approach can improve patient outcomes, long-term survival remains limited, emphasizing the need for novel strategies to control intra-abdominal disease recurrence 1. In this context, immunotherapy has emerged as a promising alternative to enhance tumor control and prevent disease progression 2.

The omentum, a specialized visceral adipose tissue, plays a crucial role in the progression and potential regression of PC. It harbors lymphoid aggregates known as milky spots (MSs), which facilitate peritoneal immune surveillance by capturing antigens, particulates, and pathogens 3. However, tumor cells exploit this immune niche by interacting with omental adipocytes and fibroblasts, leading to enhanced tumor proliferation 4. Recent preclinical studies suggest that harnessing the immune potential of the omentum through immunotherapy could transform it into an ally against PC and its primary tumors 5.

Adoptive cell therapy (ACT) is a highly personalized immunotherapeutic approach that involves the administration of immune cells with direct anticancer activity 6, 7. While most studies rely on viral vectors for T-cell modification, *in vitro*-transcribed (IVT) mRNA has emerged as an alternative, offering more cost-effective, faster manufacturing, easier scalability, enhanced safety, efficient protein translation, and controlled pharmacokinetics compared to conventional technologies 8.

The therapeutic potential of ACT has been demonstrated in multiple models, including PC. For example, mRNA electroporation has been used to engineer T cells transiently to produce single-chain IL-12, leading to significant tumor rejection upon intratumoral injection 9. Similarly, engineering tumor-infiltrating lymphocytes (TILs) or CAR T cells with IL-12/DRIL18 mRNAs has been shown to increase their antitumor efficacy 10. Locoregional adoptive transfer of transiently IL-12-armed T cells further supports the feasibility of mRNA-engineered ACT in treating PC 11.

Given the immunomodulatory potential of cytokines, particularly in the context of PC, combining ACT with immune-boosting cytokines may offer increased therapeutic benefits 12. In this context, interleukin-33 (IL-33) has gained increasing attention for its role in shaping antitumor immunity.

IL-33, a member of the IL-1 cytokine family, is constitutively expressed in epithelial and endothelial cells and plays a critical role as an alarmin upon tissue damage. It exerts its effects by binding to the ST2 receptor, leading to the activation of diverse immune subsets, including Th1 CD4^+^ T cells, CD8^+^ T cells, NK cells, dendritic cells, eosinophils, and innate lymphoid cells 13-15.

IL-33 has been explored as a therapeutic agent in several tumor models. In the context of metastatic peritoneal malignancies, IL-33, when delivered locally as recombinant protein (rIL-33), has been shown to generate a proinflammatory tumor environment, suggesting a novel treatment approach for advanced ovarian cancer 16. In peritoneal metastases of gastric cancer, local administration of rIL-33 has been reported to reshape the tumor microenvironment (TME) by reprogramming tumor-associated macrophages (TAMs), ultimately improving treatment efficacy 17. Furthermore, an engineered IL-33 activates group 2 innate lymphoid cells (ILC2s) in pancreatic cancer, leading to the formation of tertiary lymphoid structures (TLSs), which enhance anti-tumor immunity 18.

The integration of IL-33 into ACT strategies has yielded promising results. CAR-T cells transduced to co-express IL-33 and an engineered IL-2 superkine have been shown to enhance antitumor immunity in solid tumor models by altering immune cell proportions in the TME and promoting the recruitment and activation of endogenous tumor-specific T cells 19. Moreover, orthogonal gene engineering approaches incorporating IL-33 with an IL-2 variant have been found to induce an effector state in the transduced T cells employed for the ACT improving their persistence and function 20.

Considering the therapeutic potential of IL-33 and ACT, we hypothesized that electroporating tumor-specific T cells with IL-33 mRNA for transient expression alone or IL-33 mutein mRNA in combination with IL-12 mRNA could be particularly beneficial for treating tumors in the peritoneal cavity, given the unique immunological landscape of the peritoneal environment 11. This study aims to evaluate the pre-clinical evidence of this strategy in the context of PC, focusing on its impact on immune cell recruitment, tumor regression, and overall survival in murine PC models.

## Methods

### Cell lines and culture conditions

The B16.OVA melanoma line was obtained from the laboratory of Lieping Chen (Yale University, New Haven, CT), who kindly made it available for this study, while the B16.F10 cell line was obtained from ATCC. Cell lines were grown in RPMI 1640 medium containing GlutaMAX (Gibco, Waltham, MA) and 10% fetal bovine serum (Sigma-Aldrich, St. Louis, MO), 100 IU/mL penicillin, 100 µg/mL streptomycin (Gibco), and 50 µM 2-mercaptoethanol (Gibco). Selection was performed using 0.4 mg/mL G418 (Sigma-Aldrich). The Panc02-OVA cell line, kindly supplied by Sebastian Kobold (University of Munich, Germany), was cultured in high-glucose DMEM (Thermo Fisher, Hennigsdorf, Germany) supplemented in the same manner as the complete RPMI medium. The cell line MC38.CEA.Luc, that expresses the carcinoembryonic antigen (CEA) and luciferase (Luc), was cultured in complete RPMI with 0.4 mg/mL G418 (Sigma‒Aldrich) and 7 µg/mL of Puromycin (GIBCO). All cell lines underwent periodic screening for mycoplasma using the MycoAlert detection kit (Lonza, Basel, Switzerland).

### mRNA synthesis by *in vitro* transcription

UniProt was used to obtain the sequence for mature IL-33. The amino acid sequences corresponding to firefly luciferase and the single-chain IL-12 construct had been reported and characterized previously.^22^ Codon-optimized murine sequences were later synthesized and assembled by GenScript (Nanjing, China) into a pUC57 plasmid carrying an ampicillin selection marker. Upstream of the ORF's start codon, the construct contains a T7 promoter followed by a Kozak consensus sequence. Upstream mature IL-33 sequences, we added the following signal peptide: MDWTWILFLVAAATRVHS (IgE leader sequence). The stop codon is followed by two consecutive β-globin 3' UTRs and a poly(A) tail of 90-120 nucleotides. The integrity of each construct was confirmed by Sanger sequencing and by digestion with two independent restriction enzymes.

For *in vitro* transcription, the plasmids were subsequently linearized using HindIII and purified via phenol:chloroform:isoamyl alcohol extraction. *In vitro* transcription (IVT) was performed with the T7 mScript_Standard mRNA Production System (Cellscript, Madison, WI, USA). The purified mRNA was stored at -80 °C.

### Animal handling

Female C57BL/6 mice, aged six to eight weeks, were obtained from Harlan Laboratories (Barcelona, Spain) for use in the *in vivo* studies. Animals were maintained in specific pathogen-free facilities at the Cima Universidad de Navarra (Pamplona, Spain). The Ethics Committee of the University of Navarra approved the experimental protocols (063-21, and 059-23). ST2^-/-^ mice were provided by Prof. Daniel D. Pinschewer (University of Basel, Switzerland), and MHC double-knockout (dKO) mice were kindly provided by Dr. Miguel F. Sanmamed (Clínica Universidad de Navarra, Spain) 21.

For adoptive cell therapy, 5 × 10^5^ Panc02.OVA or B16.OVA or 1 x 10^6^ MC38.CEA.Luc tumor cells were injected intraperitoneally (i.p.) in 300 μL of PBS. In the rechallenge experiment, B16.F10 cells (5 × 10^5^) were injected subcutaneously (s.c.). The mice received i.p. injections of 2.5 × 10^6^ electroporated T cells on days 6 and 9 post-tumor inoculation and 2.5 × 10^6^ CAR-T cells 3 days after tumor administration.

Mice with intraperitoneal tumors were monitored for ascites development, the first detected sign of intraperitoneal malignancy, and euthanized upon signs of pain and distress. Tumor size was monitored on three separate occasions each week in s.c. models. Euthanasia was performed following approved ethical procedures.

### CAR T cell production

CD8^+^ T cells were isolated from splenocytes of C57BL/6 female mice using Miltenyi Biotec isolation kit (REF: A321875034) and following the manufacturer's recommendations. Isolated cells were activated for 48 h using mouse T-activator dynabeads CD3/CD28 (GYBCO by ThermoFisher Scientific). After that, two cycles of spinfections were performed with retrovirus carrying the CAR against CEA following the protocol as previously described 22. Electroporated cells were allowed to recover in an incubator in complete RPMI containing 50 U/mL recombinant human IL-2 (Proleukin, Novartis).

### mRNA electroporation

OVA_(257-264)_-specific CD8^+^ T cells and gp100-specific CD8^+^ T cells were isolated from the spleens of OT.I and PMEL-1 transgenic mice, respectively, as previously described 9, 11. Activated OT.I and PMEL-1 T cells were washed and then placed in OPTI-MEM without phenol-red (Gibco) at 100 × 10^6^ cells/mL. The cells (2 × 10^7^) were electroporated with 20 μg of mRNA (1 μg of mRNA per million T cells) via the Gene Pulser Mx System (Bio-Rad, Hercules, CA, USA). When co-electroporation of two different mRNAs was performed, 1 μg of mRNA per million T cells per mRNA type was used. The electroporated cells were cultured in complete RPMI medium with 50 IU/mL rhIL-2. Trypan blue staining was used to assess cell viability, and flow cytometry was used to confirm the transfection efficiency 24 h after electroporation.

### Sample processing

A total of 3 mL of chilled PBS was introduced into the peritoneal cavity to collect peritoneal lavage samples. The omentum and spleen were also harvested. Splenocytes were processed via a 70 µm cell strainer and centrifuged at 300 × g for 10 min. Red blood cells were lysed with ACK lysis buffer. Single-cell suspensions were analyzed via flow cytometry or ELISpot. Peritoneal lavage samples were centrifuged at 300 × g for 5 min and the supernatant was stored at -80 °C for further analysis. Omentum tissues were digested in PBS containing 0.075% collagenase IV (Sigma-Aldrich) for 10 minutes at 37 °C, filtered, and used for further analysis.

### Cytokine analyses

IL-33 and IFN-γ protein levels were measured via ELISA (R&D Systems, BD OptEIA). IFN-γ-secreting cells were assessed using an ELISpot assay (BD Biosciences). Splenocytes were stimulated with OVA, OVA_(257-264)_ peptide (Invivogen, San Diego, USA), or irradiated Panc02 or Panc02.OVA cells. ELISpot plates were processed via a CTL ImmunoSpot automated counter.

### Flow cytometry

Single-cell suspensions were stained with a Zombie NIR Fixable Viability Kit (BioLegend) and incubated with FcR-Block (anti-CD16/32). The following antibodies (BioLegend) were used for surface staining: PerCP-CD45 (# 103130), PE-Cy7-CD3 (# 100220), BV650-CD11b (# 101239), APC-CD11c (# 17011482), FITC-MHC-II (# 553551), PE-Siglec-F (# 12170280), BV510-Ly6G (# 127633), AF700-CD19 (# 115527), BV605-TCRβ (# 562840), PE/Dazzle-NK1.1 (# 54108747), BV510-ICOS (# 313525), APC-ST2 (# 146604), FITC-CD107a (# 121605) and PE-CD90 (# 202523). For intracellular staining, the cells were permeabilized with BD Cytofix/Cytoperm and stained with PE-IL-33 (# MA5-23640), PE-IFN-γ (# 505808) and BV421-TNF-α (# 506327). Data acquisition was performed on a CytoFLEX flow cytometer (Beckman Coulter), and analyses were conducted via FlowJo software.

### RNA isolation, RNA-seq, and bioinformatics

Total RNA was extracted from omentum samples via the RNeasy Mini Kit (Qiagen). RNA integrity was assessed via the Agilent 2200 TapeStation. The samples were sent to Macrogen, Inc., for library preparation and sequencing on a NovaSeq 6000 (Illumina).

Data preprocessing was performed as follows: first data was trimmed for adapters (Trimmomatic v0.39), aligned to the reference mouse genome (STAR v2.7.9a, ref. mm39, gencode vM27), and quantified using featureCounts (v1.6.3). Exploratory data analysis was conducted in R (v 4.4.1), through Bioconductor (v 3.19) for all the packages relevant to the analysis. Differential gene expression analysis was done via edgeR. Gene Set Enrichment Analysis (GSEA) was subsequently performed using clusterProfiler. Pathways analyzed come from a combination of Hallmark, Gene Ontology (GO), Biocarta, KEGG, PID, Reactome, and WikiPathways gene sets obtained from msigdb using R package msigdbr. For all plots ggplot2 & enrichplot were used. Top pathways were selected from the top 40 GO:BP, ranked by Normalized Enrichment Score (NES) and by removal of redundant and irrelevant pathways with no relation to the study. The raw data are available in the GEO database under accession number GSE291258.

### *In vitro* cytotoxicity assays (xCELLigence)

Real-time killing assays were conducted via an xCELLigence Real-Time Cell Analysis Instrument (ACEA). B16.OVA cells (5 × 10^5^) were plated for adherence. PMEL-1 T cells (1 × 10³) were cocultured at a 1:5 effector-to-target ratio. Electric impedance was recorded every 5 min for 42 h.

### Luminescence detection

MC38.CEA.Luc tumor cell line intraperitoneal growth follow-up was performed by bioluminescence measurement using *In Vivo Imaging System* (IVIS). Following anesthesia, the animals received an intraperitoneal injection of 100 µL luciferin (20 mg/mL; Promega, Madison, WI, USA) administered at the designated time points after tumor implantation. Bioluminescent signals were recorded following luciferin injection, and the resulting images were processed using the M3 Vision software.

### Statistical analysis

Statistical analyses were conducted via GraphPad Prism 8.2.1 and were performed using one- or two-way ANOVA followed by Sidak or Tukey post hoc testing. Survival curves were compared using the log-rank test, and differences with p values below 0.05 were regarded as statistically significant. The figure legends specify the statistical tests used in each analysis.

## Results

### CD8^+^T cells electroporated with mRNA-IL-33 exhibit increased IFN-γ expression

**Figure 1:**
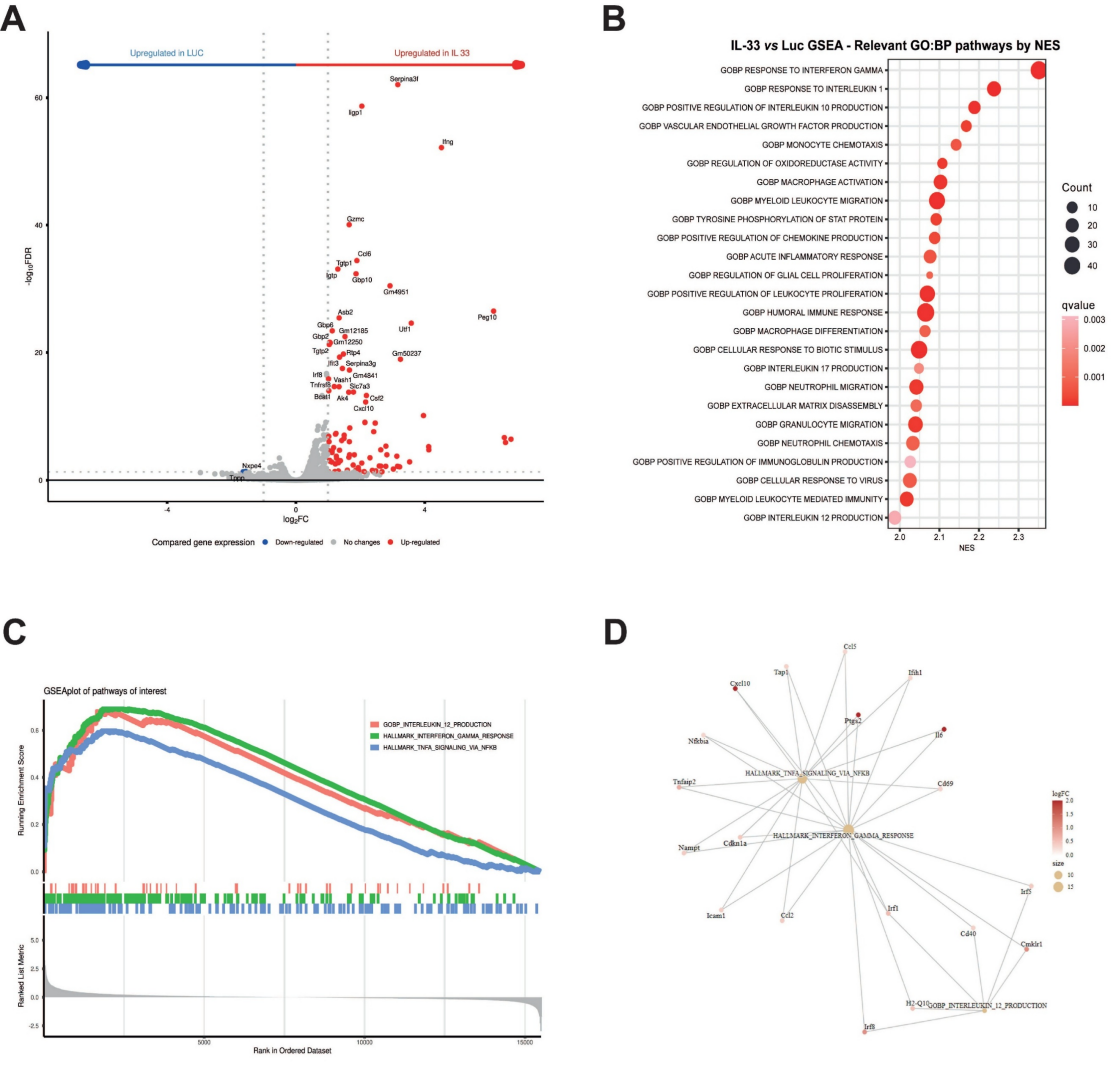
** RNA sequencing analysis of gene expression modulation in OT.I mRNA-Luc and OT.I mRNA-IL-33 T cells.** OT.I T cells were electroporated with mRNA-Luc or mRNA-IL-33 (1 µg/million cells), and total RNA was extracted 24 h post-electroporation for transcriptomic analysis by RNA sequencing (RNA-seq). (**A**) Volcano plot displaying differentially expressed genes in mRNA-Luc and mRNA-IL-33 T cells. Genes with a false discovery rate (FDR) < 0.05 are highlighted in red (upregulated) and blue (downregulated). (**B**) Gene Set Enrichment Analysis (GSEA) illustrating the top 25 significantly upregulated and downregulated Gene Ontology Biological Process (GO:BP) terms (adjusted p value < 0.05). (**C**) GSEA plot of pathways of interest to reflect the effect of IL-33 stimuli over them. (**D**) Pathway-gene linked node plot reflecting common genes across these pathways of interest.

To characterize the gene expression profile activated by IL-33 in OT.I T cells, we conducted RNA sequencing (RNA-*seq*) analysis. Total mRNA was extracted from OT.I cells 24 h post-electroporation. As depicted in the volcano plot (**Figure 1A**), IFN-γ was among the most upregulated genes in the OT.I mRNA IL-33 condition, compared with OT.I electroporated with an irrelevant mRNA encoding luciferase (OT.I mRNA-Luc). Furthermore, Gene Ontology (GO) analysis revealed the upregulation of cellular signaling pathways associated with type I and II interferons and TNF (**Figure 1B**, **C,** and **D**). Our results indicate that IL-33 produced by OT.I cells can act in an autocrine manner within T cells, inducing a proinflammatory response characterized by the release of cytokines such as IFN-γ.

**Figure 2:**
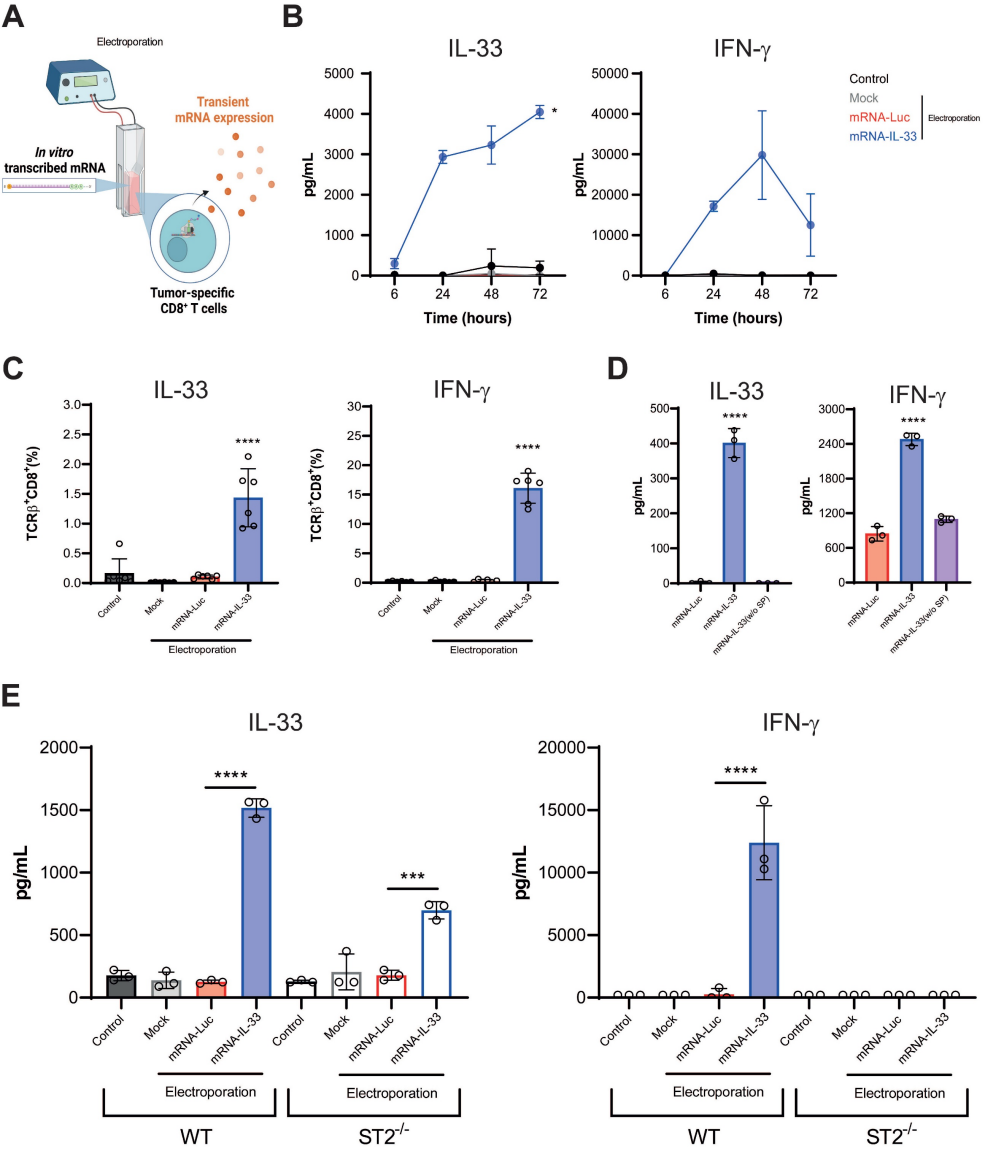
***In vitro* characterization of OT.I IL-33 T cells. (A)** OT.I T cells were activated and electroporated with either Luc or IL-33 mRNA. Murine splenocytes were also preactivated and electroporated following the same protocol. Transfection efficiency and cytokine secretion were evaluated using flow cytometry and ELISA. (**B**) Kinetics of IL-33 and IFN-γ secretion in supernatants of *in vitro* activated OT.I T cells electroporated with Luc or IL-33 mRNA, measured by ELISA at the indicated time points. Data represent biological triplicates. (**C**) Intracellular detection of IL-33 and IFN-γ in TCRβ^+^/CD8^+^ OT.I T cells 24 h post-electroporation, analyzed by flow cytometry. Data represent biological sextuplicates. (**D**) Comparison of IL-33 and IFN-γ secretion in supernatants of OT.I T cells electroporated with standard IL-33 mRNA or IL-33 mRNA lacking a signal peptide, measured 24 h post-electroporation. (**E**) IL-33 and IFN-γ production in electroporated splenocytes. Murine splenocytes were preactivated overnight with plate-bound anti-CD3 (2 µg/mL) and soluble anti-CD28 (1 µg/mL), then expanded with rhIL-2 for 48 h before electroporation with 1 µg of mRNA per million of T cells. Supernatants were collected after 24 h and analyzed by ELISA. Additionally, ST2 KO splenocytes electroporated with IL-33 mRNA were assessed for IL-33 and IFN-γ production and compared to naïve splenocytes. Statistical analysis: One-way ANOVA followed by Tukey's multiple comparison test was applied to panels **C**, **D,** and **E**. ELISA assays in **D**, and **E** were carried out twice by triplicate. Data in panel **B** are expressed as the mean ± SD and analyzed using repeated-measures ANOVA. *p < 0.05; ***p < 0.001, ****p < 0.0001.

To further validate these findings, we performed flow cytometry and ELISA assays (**Figure 2A**). Kinetic analysis of transient protein expression in the supernatants of OT.I mRNA-IL-33 cells confirmed that IL-33 and IFN-γ were produced (**Figure 2B**). Additionally, flow cytometry analysis 24 h post-electroporation confirmed the presence of intracellular IL-33 and IFN-γ in the OT.I T cells (**Figure 2C**). To demonstrate that IL-33 autocrine activity requires secretion into the extracellular space, we generated mRNA encoding IL-33 without the signal peptide. This approach completely abrogated IL-33 activity in terms of IFN-γ secretion (**Figure 2D**). Interestingly, when splenocytes from transgenic mice deficient for IL-33 receptor (ST2^-/-^) were electroporated with IL-33 mRNA, IFN-γ was not detected in the supernatants 24 h later, indicating that IL-33 signaling through ST2 is required for IFN-γ induction (**Figure 2E**).

### The ACT-mediated antitumor effect combined with IL-33 expression is intrinsic to ST2 expression in transferred lymphocytes and triggers an antigen-specific immune response

**Figure 3:**
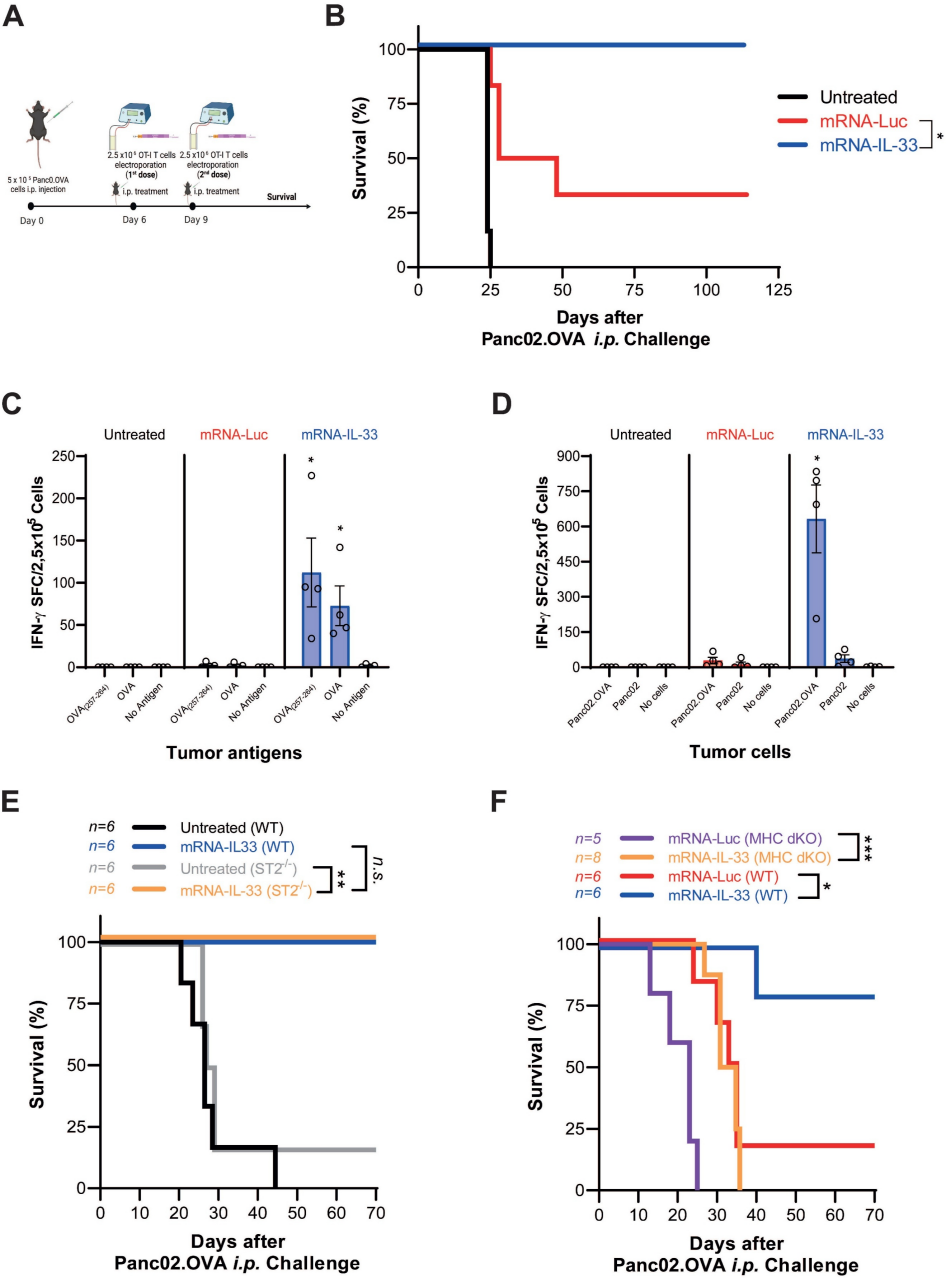
** The ACT-mediated antitumor effect combined with IL-33 in a peritoneal carcinomatosis model derived from Panc02.OVA cancer cells triggered an antigen-specific immune response.** (**A**-**B**) Survival analysis of treated mice. C57BL/6 mice were injected intraperitoneally (i.p.) with 5 × 10^5^ Panc02.OVA tumor cells. On days 6 and 9 post-tumor inoculation, the mice received 2.5 × 10^6^ OT.I T cells electroporated with either Luc or IL-33 mRNA. Survival was monitored over time in three groups: untreated group, the OT.I mRNA-Luc group, and OT.I mRNA-IL-33 (n = 6 per group). (**C-D**) Evaluation of IFN-γ-producing splenocytes. Fifteen days after tumor inoculation, the mice (n = 4 per group) were sacrificed, and the splenocytes were analyzed via an ELISpot assay for IFN-γ production, following *ex vivo* stimulation. The cells were stimulated either with OVA protein, (**C**) SIINFEKL peptide (OVA_257-264_) ; (**D**) irradiated Panc02 tumor cells (2.5 × 10^4^, 20,000 rads), or irradiated Panc02.OVA tumor cells (2.5 × 10^4^, 20,000 rads). (**E**) Survival analysis of wild-type (WT) and ST2^-/-^ mice. C57BL/6 WT (n = 6 per group) and ST2^-/-^ mice (n = 6 per group) were injected i.p. with 5 × 10^5^ Panc02.OVA tumor cells. Electroporated OT.I T cells (2.5 × 10^6^) were administered i.p. on days 6 and 9 post tumor inoculation. Kaplan-Meier survival curves are shown. (**F**) Assessment of IL-33-mediated antitumor activity in immunodeficient mice. MHC double-knockout (MHC dKO) mice or wild-type mice were injected i.p. with 5 × 10⁵ Panc02.OVA tumor cells and treated with OT.I T cells electroporated with Luc or IL-33 mRNA following the same schedule as above. Kaplan-Meier survival curves are shown. Statistical analysis: The log-rank (Mantel‒Cox) test was used for survival data in panels (**A**), (**E**), and (**F**). The statistical significance in panel (**C**-**D**) was determined via two-way ANOVA followed by Tukey's multiple comparisons test. *p < 0.05; **p < 0.01; ns: non-significant. Data are representative of two independent experiments.

After *in vitro* characterization, OT.I mRNA-IL-33 cells were used for adoptive cell therapy in a murine PC model derived from Panc02.OVA pancreatic cancer cell line stably transduced to express ovalbumin (OVA), whose epitope is recognized by OT.I in the context of H2Kb*.* The animals received an intraperitoneal (i.p.) injection of 5 × 10^5^ Panc02.OVA tumor cells to establish the PC model. On days 6 and 9 post-tumor inoculation, the mice received two doses of 2.5 × 10^6^ OT.I mRNA-Luc or -IL-33 cells i.p. (**Figure 3A**). This treatment resulted in tumor rejection and significantly improved survival, with 100% of the mice remaining tumor-free at the end of the follow-up period (**Figure 3B**). To determine whether IL-33-based ACT induced a systemic tumor-specific response, an ELISpot analysis was performed on splenocytes collected six days after the last infusion. A strong IFN-γ response was detected upon stimulation with OVA protein, the OVA₂₅₇-₂₆₄ peptide, or irradiated Panc02.OVA cells, but not with parental Panc02 cells (**Figure 3C-D**). In addition, IL-33 enhanced the persistence of transferred OT.I cells in peripheral blood (CD45.1⁺) and promoted the expansion of endogenous OVA-specific CD8⁺ T cells (CD45.2⁺) (**Figure S1**).

To further determine whether the antitumor effect of IL-33 is intrinsic to the engineered T cells or influenced by host immune components, we performed adoptive transfer experiments in both wild-type (WT) and ST2⁻^/^⁻ mice, as well as in immunodeficient MHC double-knockout (dKO) mice. As shown in **Figure 3E**, both WT and ST2⁻^/^⁻ mice effectively controlled pancreatic peritoneal tumors after ACT with OT.I mRNA-IL-33 cells, indicating that IL-33 signaling in host cells is not essential for therapeutic efficacy. Moreover, in **Figure 3F**, IL-33-engineered OT.I T cells also extended survival in immunodeficient mice, confirming that IL-33 confers a cell-intrinsic antitumor advantage. However, the overall effect was more pronounced in WT hosts, suggesting that interactions with endogenous immune populations can further enhance the therapeutic benefit of IL-33-expressing T cells.

### Intraperitoneal delivery of IL-33 mRNA via adoptive transfer modulates the TME

**Figure 4:**
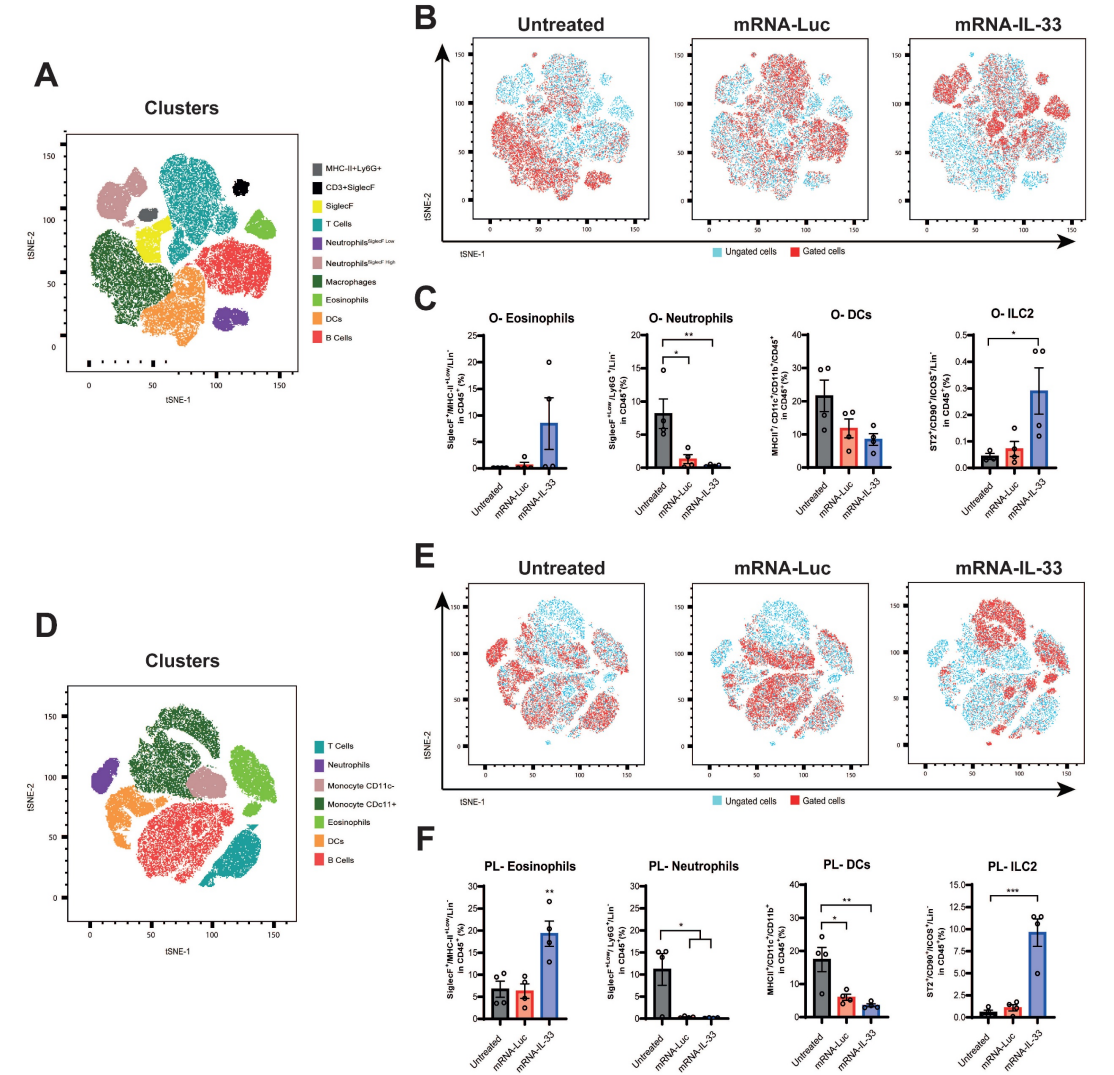
**Intraperitoneal delivery of IL-33 mRNA via adoptive transfer modulates the tumor microenvironment.** Mice (n = 4 per/group) were treated as described in **Figure 3A-B**. Six days after the second treatment (day 15 after tumor challenge), the mice were sacrificed, and peritoneal lavage samples and omentum were collected for immune profiling via flow cytometry. (**A-B**) t-SNE plots illustrating immune cell distribution in the omentum (O). (**C**) Percentages of the different immune cell populations in the omentum are shown. (**D-E**) t-SNE analysis was used to evaluate differences in the immune cell populations in peritoneal lavage samples (PL). Immune populations were defined as CD3^+^, T cells; CD11b^+^ Ly6G^+^ SiglecF^+Low^, neutrophils (SiglecF^Low^); CD11b^+^ MHC-II^+^ CD11c^+^ Ly6G^+^ SiglecF^+High^, neutrophils (SiglecF^High^); CD11b^+^ MHC-II^+^ CD11c^+^ Ly6G^-^, macrophages; MHC-II^+^ Ly6G^+^ SiglecF^+^, eosinophils; MHC-II^+^ CD11c^+^ CD11b^+^, dendritic cells (DCs); and CD19^+^ MHC-II^+^ Ly6G^-^, B cells. (**F**) Percentages of the different immune cell populations in the peritoneal lavage samples are displayed. Statistical significance was determined by two-way ANOVA in panel **C** and **F**. *p < 0.05; **p < 0.01; ***p < 0.001. Data are representative of two independent experiments.

IL-33 has pleiotropic effects on different immune cell types 13, 15. To investigate how IL-33-expressing-OT.I T cells modulate the TME, we analyzed peritoneal lavage sample and omentum six days after the last treatment (day 15). As shown in the t-SNE plots, OT.I mRNA-IL-33 compared with the control treatment led to distinct immune cell distributions in the omentum (**Figure 4A-C**) as well as in the peritoneal cavity (**Figure 4D-F**). These changes were confirmed by manual flow-cytometry analysis (**Figure S2 and S3**). Flow cytometric profiling revealed that ILC2s (Lin^-^ TCRβ^-^ CD3^-^ CD19^-^ NK1.1^-^ CD11c^-^ ICOS^+^ CD90^+^ ST2^+^) and eosinophils (Lin^-^ CD11b^+^ SiglecF^+^) were significantly enriched in the omentum and peritoneal lavage samples from the OT.I mRNA-IL33-treated mice, whereas neutrophil populations decreased (**Figure 4C and F**). Lymphoid and myeloid populations were also analyzed, as depicted in **Figure S4**. A marked decrease in the myeloid population (CD45^+^ TCRβ^-^ CD19^-^ CD11b^+^) was observed in both OT.I mRNA-Luc and OT.I mRNA-IL-33-treated groups in the omental tumor microenvironment and peritoneal cavity (**Figure S4C and F**). In omental tissues collected from animals administered OT.I mRNA-IL-33 cells, a significant increase in the number of NK cells (CD45^+^ TCRβ^-^ CD19^-^ NK1.1^+^) was observed (**Figure S4C**). To complement the flow cytometry data, we performed RNA-seq analysis of omentum tissue (**Figure S5**). The first panel shows a marked reduction in omentum weight in mice treated with OT.I mRNA-IL-33, indicating that most tumor cells had already been cleared at this time point. Transcriptomic profiling further revealed an ongoing inflammatory and interferon-driven response in the OT.I mRNA-Luc group, whereas the IL-33 group displayed a gene-expression pattern consistent with tissue normalization after tumor elimination. Taken together, the flow cytometry and RNA-seq data demonstrate that IL-33 not only accelerates tumor clearance but also induces a profound remodeling of the TME, transitioning it from an inflamed, tumor-bearing state to an immune-resolved, tumor-free environment.

### IL-33 mutein enhances the antitumor effect on a PC model

The half-life of IL-33 in its active form is limited by the oxidation of its disulfide bonds, which leads to its inactivation 23. To improve IL-33 stability and functionality, we generated an IL-33 mutein sequence with four cysteine-to-serine substitutions, preventing disulfide bond formation, which inhibits ST2 receptor binding.

**Figure 5:**
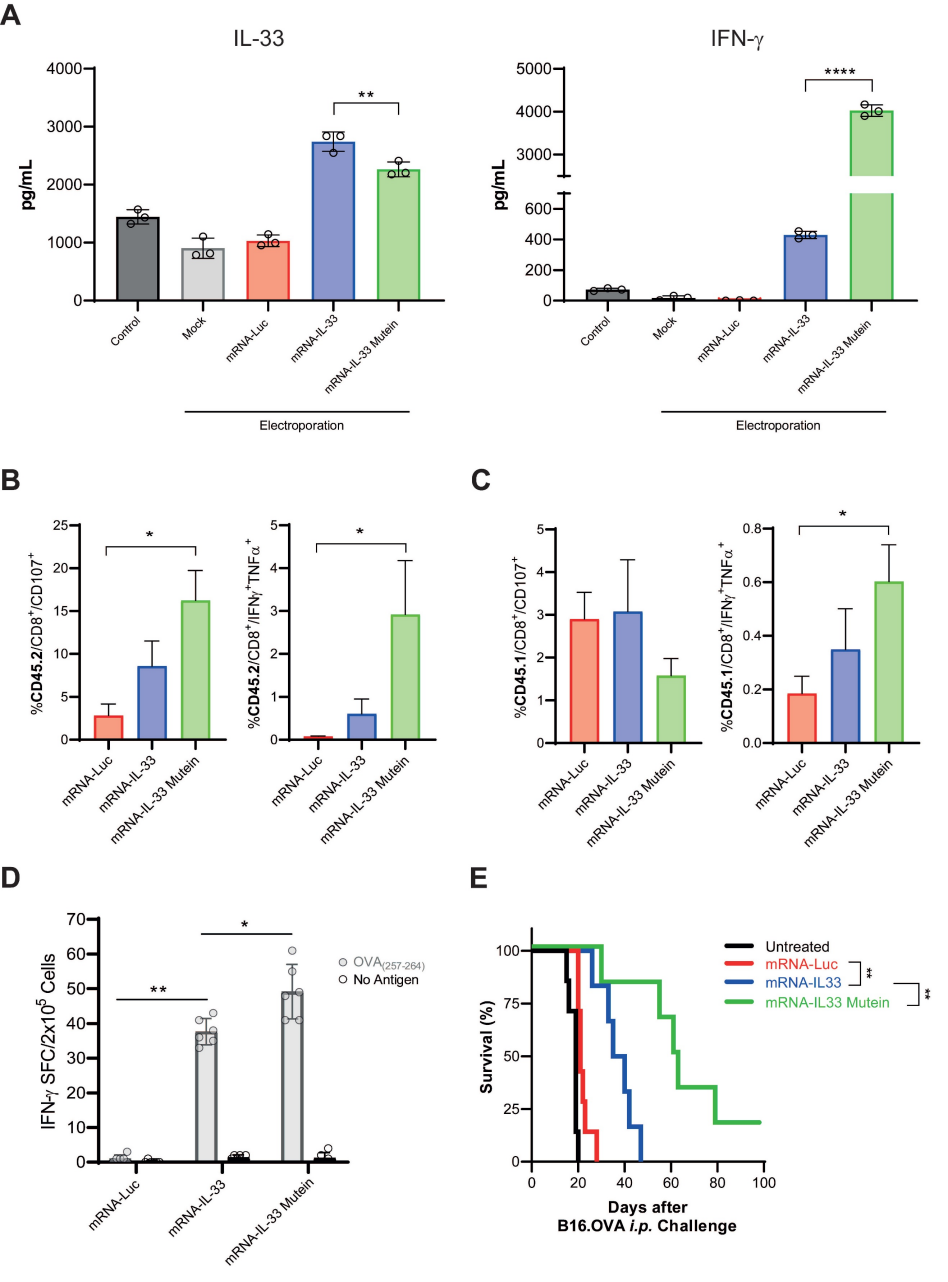
** IL-33 mutein mRNA-electroporated CD8^+^ T cells enhance the antitumor effect in an aggressive PC model.** (**A**) IL-33 and IFN-γ production was analyzed by ELISA 24 h after the electroporation of OT.I T cells with Luc, IL-33 or IL-33 mutein mRNAs. (**B, C**) C57BL/6 CD45.2 mice were injected intraperitoneally (i.p.) with 2 × 10^5^ B16.OVA tumor cells (n = 4 per/group). On day 6 post-tumor inoculation, mice received 2.5 × 10^6^ CD45.1 OT.I T cells electroporated with Luc, IL-33, or IL-33 mutein mRNAs. Six days after treatment, the omentum was collected and analyzed for (i) degranulation capacity (CD107a expression) and (ii) polyfunctionality based on the co-expression of IFN-ɣ and TNF-α. Donor (CD45.1⁺) CD8⁺ T-cell responses are shown in **B**, and endogenous (CD45.2⁺) CD8⁺ T-cell responses in **C**. (**D**) Six days after tumor inoculation, mice (n = 6 per group) were sacrificed, and CD8^+^ T cells were isolated from the spleen using magnetic beads. Antigen-specific IFN-γ production was assessed by ELISpot following *ex vivo* stimulation with the SIINFEKL peptide (OVA₂₅₇-₂₆₄). (**E**) Survival analysis of B16.OVA-bearing mice. C57BL/6 mice (n = 6 per group) received i.p. injections of 2.5 × 10^6^ OT.I T cells electroporated with Luc, IL-33, or IL-33 mutein mRNA. The survival of the treated mice was monitored over time. Statistical analysis: One-way ANOVA followed by Tukey's multiple comparisons test were applied to panel **A, B, C and D**. The log-rank (Mantel‒Cox) test was used to analyze the survival (**E**). **p < 0.01; ****p < 0.0001. Data are representative of two independent experiments.

*In vitro*, IL-33 mutein was detected at lower levels than wild-type IL-33; however, despite its reduced concentration, it induced IFN-γ expression in OT.I T cells 24 h post-electroporation at higher levels than wild-type IL-33 (**Figure 5A**). To further characterize the enhanced response mediated by the IL-33 mutein, we analyzed the polyfunctionality of both transferred CD8⁺ T cells (CD45.1⁺) (**Figure 5B**) and endogenous CD8⁺ T cells (CD45.2⁺) (**Figure 5C**) in tumor-bearing mice after ACT. The results showed that expression of the IL-33 mutein increased the proportion of CD8⁺ T cells, co-expressing IFN-γ and TNF-α, compared with wild-type IL-33, and also promoted stronger activation of endogenous CD8⁺ T cells. Consistent with these findings, ELISpot analysis revealed a higher frequency of OVA-specific IFN-γ-producing cells in the IL-33 mutein group (**Figure 5D**).

To assess its therapeutic potential, we established a B16.OVA PC model by injecting 5 × 10^5^ B16.OVA cells i.p. Mice were treated on days 6 and 9 with 2.5 × 10^6^ OT.I cells electroporated with irrelevant mRNA or either IL-33 wild-type or IL-33 mutein mRNAs (**Figure 5E**). In this model, OT.I mRNA-IL-33 cells exerted a significant antitumor effect; however, all mice succumbed by day 50 (**Figure 5E**). Interestingly, the OT.I mRNA-IL-33 mutein-treated mice presented a significantly greater survival advantage, demonstrating the increased antitumor efficacy of the IL-33 mutein (**Figure 5E**).

### IL-33 mutein mRNA and IL-12 mRNA synergistically enhance the antitumor efficacy of intermediate-affinity TCR-bearing CD8^+^ T cells in a PC model

To further enhance the antitumor effect, we investigated the synergistic combination of IL-33 (either WT or mutein) and IL-12 mRNAs. Given that OT.I TCR has high affinity, we tested this combination in PMEL-1 T cells, which carry an intermediate-affinity TCR specific for gp100, a melanocytic protein.

**Figure 6:**
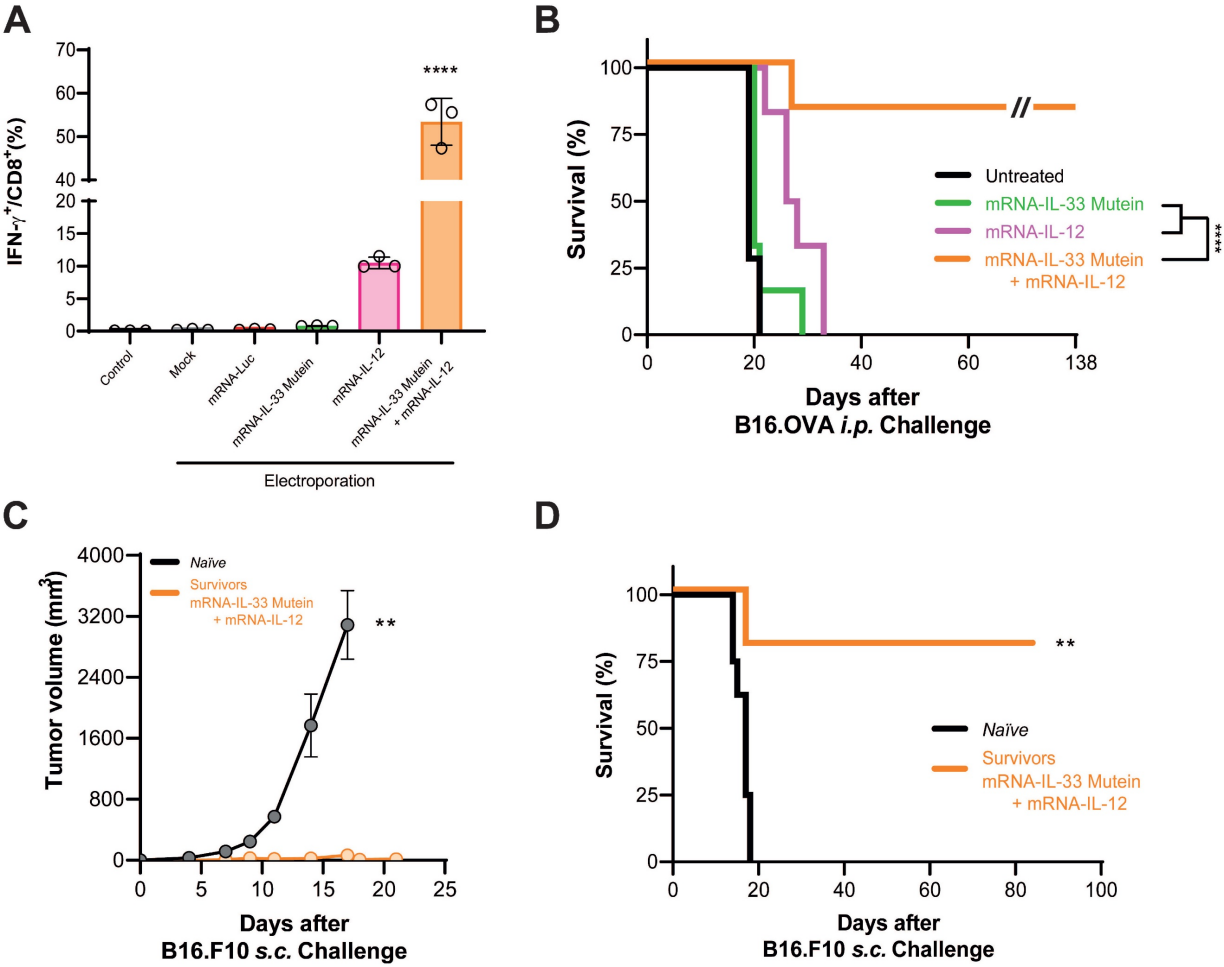
** Synergistic effect of IL-33 mutein mRNA and IL-12 mRNA electroporated in lower-affinity TCR-bearing PMEL-1 T cells in a PC model.** (**A**) 24 h after being electroporated with the corresponding mRNAs, PMEL-1 T cells were stained intracellularly with IFN-γ and subjected to flow cytometry analysis. (**B**) Survival analysis of tumor-bearing mice treated with electroporated PMEL-1 T cells. C57BL/6 mice (n = 6‒7 per group) were injected i.p. with 5 × 10^5^ B16.OVA tumor cells. On days 6 and 9, the mice received 2.5 × 10^6^ PMEL-1 T cells i.p., and survival was monitored over time. (**C-D**) B16.OVA-cured mice (n = 5) from the PMEL-1 IL-33 mutein + IL-12 treatment group (**B**) were rechallenged with a subcutaneous (s.c.) injection of parental B16.F10 cell line (5 × 10^5^ cells/mouse) on day 138. Tumor growth and survival were monitored. Statistical analysis: One-way ANOVA followed by Tukey's multiple comparisons test was applied to panel **A**. The Mann‒Whitney test was used for statistical analysis in panel **C**. Survival data were analyzed via the log-rank (Mantel‒Cox) test (**B, D**). **p < 0.01, ****p < 0.0001. Data are representative of two independent experiments.

Given the high affinity of the OT.I TCR, we evaluated this combination in PMEL-1 T cells, which express a TCR of moderate affinity specific for gp100, a melanocytic-derived antigen. Flow cytometry analysis showed that electroporation of PMEL-1 T cells with wild-type IL-33 mRNA did not significantly induce IFN-γ expression (**Figure S6A**). However, co-electroporation with both wild-type IL-33 and IL-12 mRNAs significantly increased IFN-γ expression (**Figure S6A**). Additionally, xCELLigence cytotoxicity assays demonstrated that PMEL-1 cells electroporated with IL-33 and IL-12 mRNAs were significantly more effective at killing B16.OVA tumor cells (**Figure S6B**). Nevertheless, the synergistic effect observed *in vitro* was not reproduced *in vivo* (**Figure S6C**). We then assessed whether the synergistic effect of IL-33 with IL-12 could be replicated using an IL-33 mutein mRNA. *In vitro* validation by flow cytometry revealed that co-electroporation with IL-33 mutein and IL-12 mRNAs induced a 5-fold increase in IFN-γ production (**Figure 6A**), confirming the synergistic effect. For *in vivo* validation, B16.OVA-bearing mice were treated with PMEL-1 T cells co-electroporated with IL-33 mutein and IL-12 mRNAs. These mice showed significantly improved tumor control and survival, with approximately 80% remaining tumor-free at the end of the follow-up period (**Figure 6B**). Long-term immunity was confirmed, as mice cured of B16.OVA tumors successfully rejected a subcutaneous rechallenge with parental B16.F10 cells on day 138 (**Figure 6C-D**).

**Figure 7:**
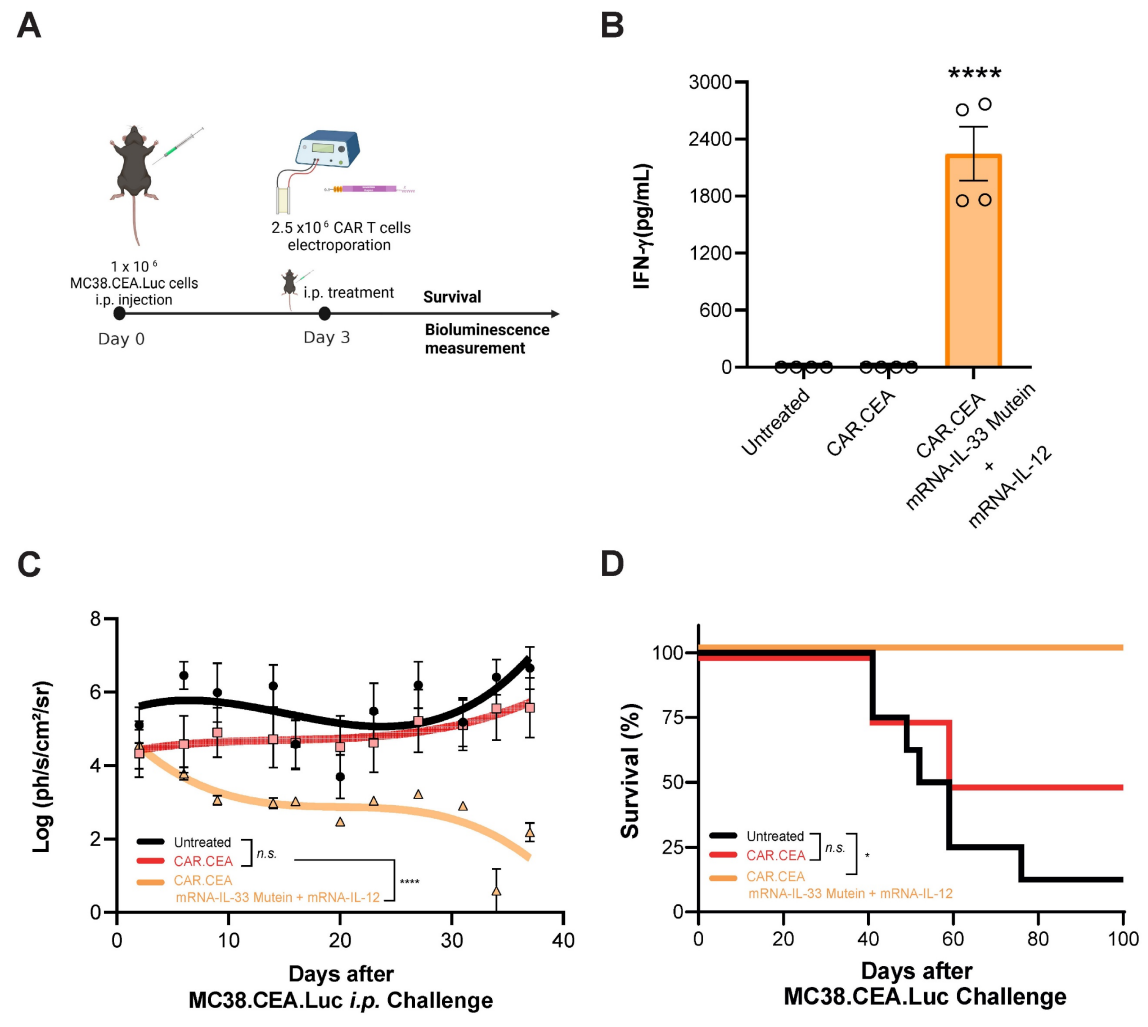
** Synergistic effect of mRNA-IL-33 mutein and mRNA-IL-12 in CAR T cells against CEA-expressing tumor cells.** (**A**) C57BL/6 mice (n = 4 per group) were injected i.p. with 1 × 10^6^ MC38.CEA.Luc tumor cells. On day 3 the mice received 2.5 × 10^6^ CAR T cells against CEA antigen i.p., and survival and luciferase expression were monitored. (**B**) IFN-γ levels in serum samples were analyzed by ELISA 4 days after the treatment with CAR T cells. (**C**) *In vivo* bioluminescence quantification (log(ph/s/cm^2^/sr)) with *in vivo* imaging system (IVIS) over-time. (**D**) Survival follow-up is represented. Statistical analysis: One-way ANOVA followed by Tukey's multiple comparisons test was applied to panel **B**. Survival data were analyzed via the log-rank (Mantel‒Cox) test (**D**). *p < 0.05, ****p < 0.0001. Data are representative of two independent experiments.

Lastly, we evaluated the synergistic effect of IL-33 mutein and IL-12 in the context of CAR T cell therapy. To this end, we established a peritoneal carcinomatosis (PC) model using MC38 tumor cells expressing human carcinoembryonic antigen (CEA) and luciferase (Luc) (**Figure 7A**). Luciferase expression enables the monitoring of intraperitoneal tumor burden via bioluminescence imaging. Mice were treated i.p. with 2.5 x 10^6^ CAR T cells 3 days after tumor inoculation (1 x 10^6^ MC38.CEA.Luc i.p.). As shown in **Figure 7B**, mice receiving CAR T cells electroporated with IL-33 mutein and IL-12 mRNAs exhibited significantly elevated serum IFN-γ levels, indicating enhanced immune activation. Additionally, luciferase signal intensity was significantly lower over time in this group compared to both untreated mice and those treated with conventional CAR T cells without cytokine mRNA expression (**Figure 7C**). Most notably, this combination therapy achieved 100% survival, in contrast to 50% survival in the group treated with CAR T cells alone (**Figure 7D**). Thus, adoptive cell therapy using CAR T cells electroporated with IL-33 mutein and IL-12 mRNAs demonstrates potent antitumor activity.

## Discussion

In this study, we demonstrated that ACT utilizing tumor-specific T cells with IL-33 mRNA significantly enhances antitumor immunity in preclinical models of PC. Our results highlight the ability of IL-33 to potentiate ACT by inducing IFN-γ expression, modulating the TME, and promoting a long-lasting immune response. Furthermore, we show that engineering T cells with an IL-33 mutein variant enhances their therapeutic efficacy and that combining IL-33 with IL-12 mRNA further amplifies the antitumor response, particularly necessary in tumor specific T cells bearing intermediate-affinity TCRs.

IL-33 is typically localized in the nucleus or cytosol because its full-length form contains a nuclear localization sequence and chromatin-binding domains that anchor the protein to histones, thereby regulating its intracellular retention and preventing uncontrolled extracellular release. Under physiological conditions, IL-33 is released as an alarmin only after cellular stress or necrosis 15. In our study, however, we used the mature IL-33 sequence preceded by a signal peptide, which directs the protein to the secretory pathway and enables its active secretion into the extracellular space. This modification bypasses the requirement for cell damage-induced release, ensuring transient and controlled availability of bioactive IL-33 following mRNA electroporation. Consequently, the cytokine can act both in an autocrine manner on the engineered T cells and in a paracrine fashion within the tumor microenvironment, enhancing antitumor activity while limiting intracellular accumulation.

Our RNA-seq analysis identified IFN-γ as one of the most upregulated genes in mRNA-IL-33 cells. A previous study reported the ability of IL-33 to promote IFN-γ induction 24; however, its direct effect on T cells in driving IFN-γ expression had not been previously described. This finding was further validated through ELISA and flow cytometry. Furthermore, using ST2 KO mice, we demonstrated that IFN-γ secretion was dependent on ST2 receptor expression in T cells. These findings suggest that IL-33 not only promotes T-cell activation but also enhances effector function through IFN-γ-mediated immune modulation. A survival advantage was observed in mice treated with mRNA-IL-33 cells in an advanced pancreatic cancer model, underscoring the therapeutic relevance of this mechanism.

In addition to its direct effects on T cells, IL-33 has pleiotropic functions in shaping the immune landscape of the TME. Our flow cytometry data revealed a significant enrichment of ILC2s and eosinophils, along with a concurrent reduction in neutrophils across peritoneal lavage fluids and omental specimens derived from the mRNA-IL-33-treated mice. This shift in immune composition suggests that IL-33 shifts the TME toward a state less permissive to tumor progression. These findings align with those of prior studies indicating that IL-33 can drive type 2 immunity, which may have dual effects on either promoting or restraining tumor growth, depending on context 25.

The immune remodeling observed in the omentum and peritoneal cavity following IL-33-based ACT suggests a coordinated response between these two interconnected anatomical compartments. The omentum is a key immune niche that harbors resident macrophages, B cells, and T cells capable of sensing and amplifying local inflammatory signals. The parallel changes detected in both tissues likely reflect dynamic migration and activation of immune subsets driven by IL-33-induced cytokine cues, which collectively contribute to the establishment of a proinflammatory tumor microenvironment. Given the elevated levels of IFN-γ detected in serum, we acknowledge the potential risk of cytokine-mediated toxicity. Nevertheless, no evidence of systemic distress or weight loss was observed in treated mice, supporting the notion that transient cytokine expression achieved through mRNA electroporation provides a favorable therapeutic window that minimizes systemic cytokine storm while maintaining potent antitumor efficacy.

Despite the promising immunomodulatory effects of IL-33, *in vivo* IL-33 can be inactivated by oxidation. To address this, we engineered an IL-33 mutein variant with four cysteine-to-serine substitutions to prevent disulfide bond formation and enhance stability 23. Our *in vitro* data demonstrated that this modified IL-33 was highly bioactive, as evidenced by its ability to induce IFN-γ expression. The enhanced bioactivity is likely due to the enhanced stability due to the oxidation resistance. The increased survival of mice treated with mRNA-IL-33 mutein T cells in the more difficult-to-treat B16.OVA PC model further supports its improved therapeutic potential.

To further increase ACT efficacy, we explored the combination of IL-33 mutein with IL-12, a cytokine known to enhance T-cell function and increase ST2 receptor expression. Our findings demonstrated that co-electroporation of PMEL-1 T cells with IL-33 mutein and IL-12 mRNAs significantly enhanced IFN-γ production and tumor cell death *in vitro*. This synergy can be attributed to the previously reported ability of IL-12 to enhance ST2 receptor expression 14, 26-28. Importantly, this synergistic effect was validated *in vivo*, where the combination of IL-33 mutein and IL-12 mRNAs resulted in superior tumor control and survival in B16.OVA PC model. Additionally, mice cured of peritoneal tumors exhibited resistance to subsequent subcutaneous B16.F10 challenge, indicating the establishment of long-term immune memory to non-dominant tumor-associated antigens. Mechanistically, IL-33 and IL-12 likely cooperate through complementary pathways: IL-12 enhances cytotoxicity and IFN-γ production via STAT4 activation, while IL-33 promotes T-cell survival and effector differentiation through ST2-mediated signaling. The combined, transient expression of both cytokines may thus synergize within the same T cell population, or across distinct immune subsets, to amplify antitumor immunity while preserving safety.

Our study provides strong evidence that engineering T cells with engineered IL-33 and IL-12 can overcome key limitations associated with ACT in solid tumors. While CAR-T cells have shown remarkable success in treating hematologic malignancies, their efficacy in solid tumors has been hindered by the immunosuppressive TME and poor T-cell persistence 29. The combination of engineered IL-33 mutein and IL-12 may help address these barriers by enhancing T-cell activation, remodeling TME, and promoting durable antitumor immunity.

Although our findings demonstrate the potential of IL-33-based ACT, several aspects warrant further investigation. The dual role of IL-33 in immunity and inflammation necessitates a deeper understanding of its effects in different tumor contexts. Moreover, as our current murine models do not fully replicate the complex stromal and immunosuppressive features of advanced solid tumors, future studies using more physiologically relevant models will be required to validate translational applicability. In addition, potential off-target effects of IL-33 must be evaluated to ensure safety in clinical settings. Further optimization of dosing regimens, delivery methods, and combinatorial strategies will be essential to advance this approach toward human ACT therapies 30.

In conclusion, this study establishes IL-33 as a potent enhancer of ACT, demonstrating its ability to promote IFN-γ expression, remodel the TME, and improve survival outcomes in PC models. The development of an engineered IL-33 variant further enhances its stability and efficacy, while coadministration with IL-12 maximizes therapeutic benefits. These findings provide a strong rationale for incorporating engineered IL-33 and IL-12 into future ACT clinical trials for solid tumor treatment.

## Supplementary Material

Supplementary figures.

## Abbreviations

WT): Adoptive cell therapy (ACT
