# Supplementary material for: Autocrine activity of engineered IL-33 mRNA enhances adoptive T-cell therapy for peritoneal carcinomatosis and synergizes with IL-12 mRNA
Source: Theranostics. 2026 Jan 1;16(7):3541–55. doi: 10.7150/thno.122132 (PMC12846726; doi:10.7150/thno.122132)
Supplement: Supplementary file 1 — Supplementary figures. [file thnov16p3541s1.pdf]

Autocrine activity of engineered IL-33 mRNA enhances adoptive T-cell therapy for peritoneal carcinomatosis and synergizes with IL-12 mRNA

Leire Arrizabalaga[1,2], Claudia Augusta Di Trani[1,2], Celia Gomar[1,2], Daniel Moreno-Luqui[1,2], Nuria Ardaiz[1,2], Virginia Belsue[1,2], José González-Gomariz[1,2], David Ruiz-Guillamon[1,2], Aline Risson[1,2], Yufei Zheng[1,2], Eduardo Huarte[1,*], Ignacio Matos[4], Ignacio Melero[1,2,3,4], Pedro Berraondo[1,2,3,#], Fernando Aranda[1,2,#]

[1]Program of Immunology and Immunotherapy, Cima Universidad de Navarra, Cancer Center Clínica Universidad de Navarra (CCUN), Pamplona, Spain.

[2]Navarra Institute for Health Research (IDISNA), Pamplona, Spain.

[3]Centro de Investigación Biomédica en Red de Cáncer (CIBERONC), Madrid, Spain.

[4]Departments of Immunology and Oncology (CCUN), Clínica Universidad de Navarra, Pamplona, Spain.

[5]Nuffield Department of Medicine (NDM), University of Oxford, Oxford, United Kingdom.

* Present address: Vaccines and immunotherapies, AstraZeneca.

# Share senior co-authorship.

Content: Supplemental Figures S1-S5

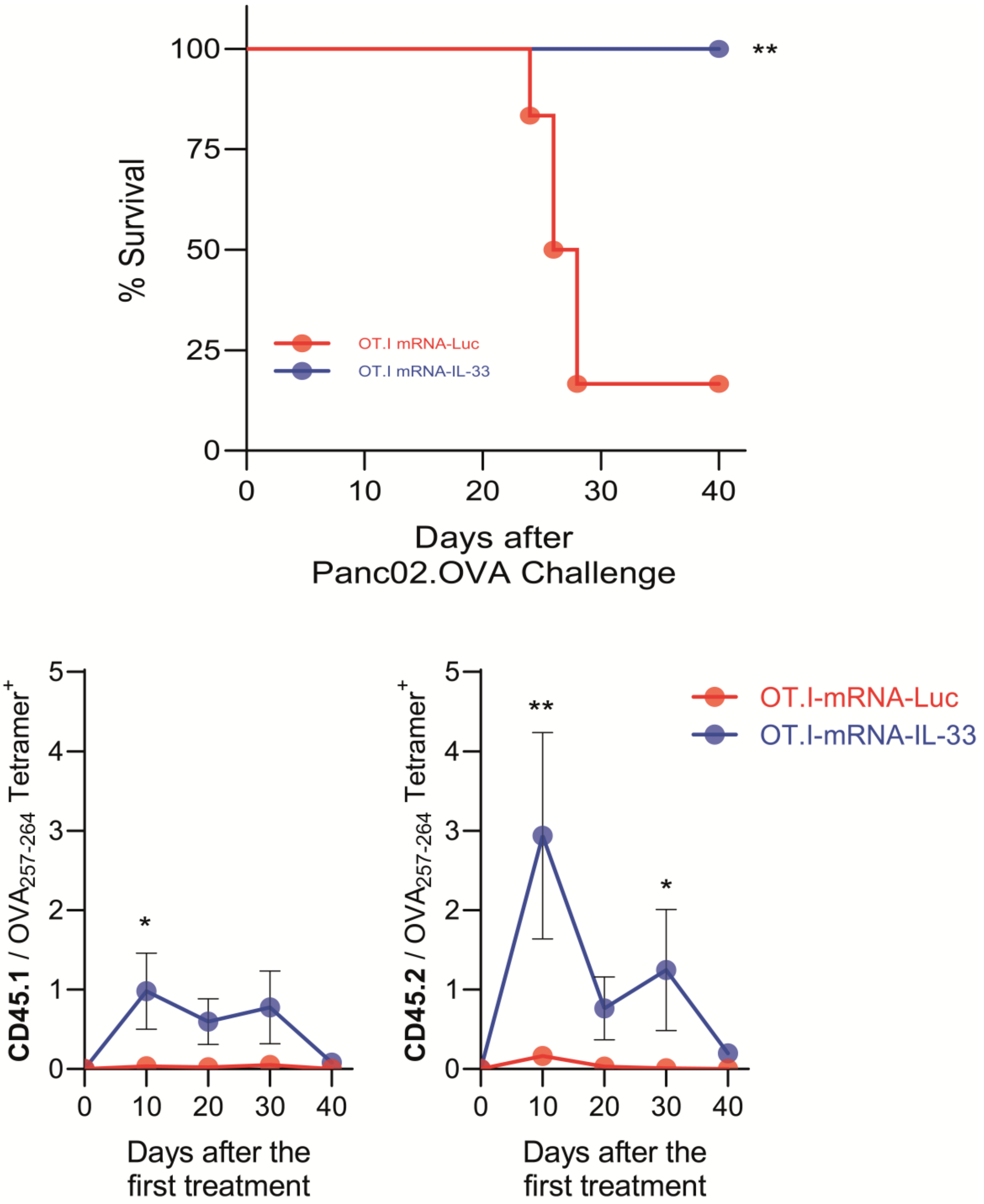

Supplementary Figure 1. IL-33 confers greater persistence of ACTs and induces an endogenous antitumor-specific $CD8^+$ T-cell response. C57BL/6 mice (n =6 per group) were injected i.p. with 5×10^5 Panc02.OVA tumor cells. On days 6 and 9 post-tumor inoculation, mice received 2.5×10^6 OT.I T cells electroporated with either Luc mRNA or

IL-33 mRNA. Ten days after the second ACT infusion, peripheral blood was collected to analyze by flow cytometry to quantify the antigen-specific $CD8^+$ T cells using $OVA_{257-264}$ tetramer staining. Both transferred (CD45.1) and endogenous (CD45.2) $CD8^+$ T-cell responses were monitored. A kinetic analysis was performed every 10 days up to day 40 after the last ACT administration. The Mann–Whitney test was used for statistical analysis and Survival data were analyzed via the log-rank (Mantel-Cox) test (panel C). *$p<0.05$, **$p<0.01$.

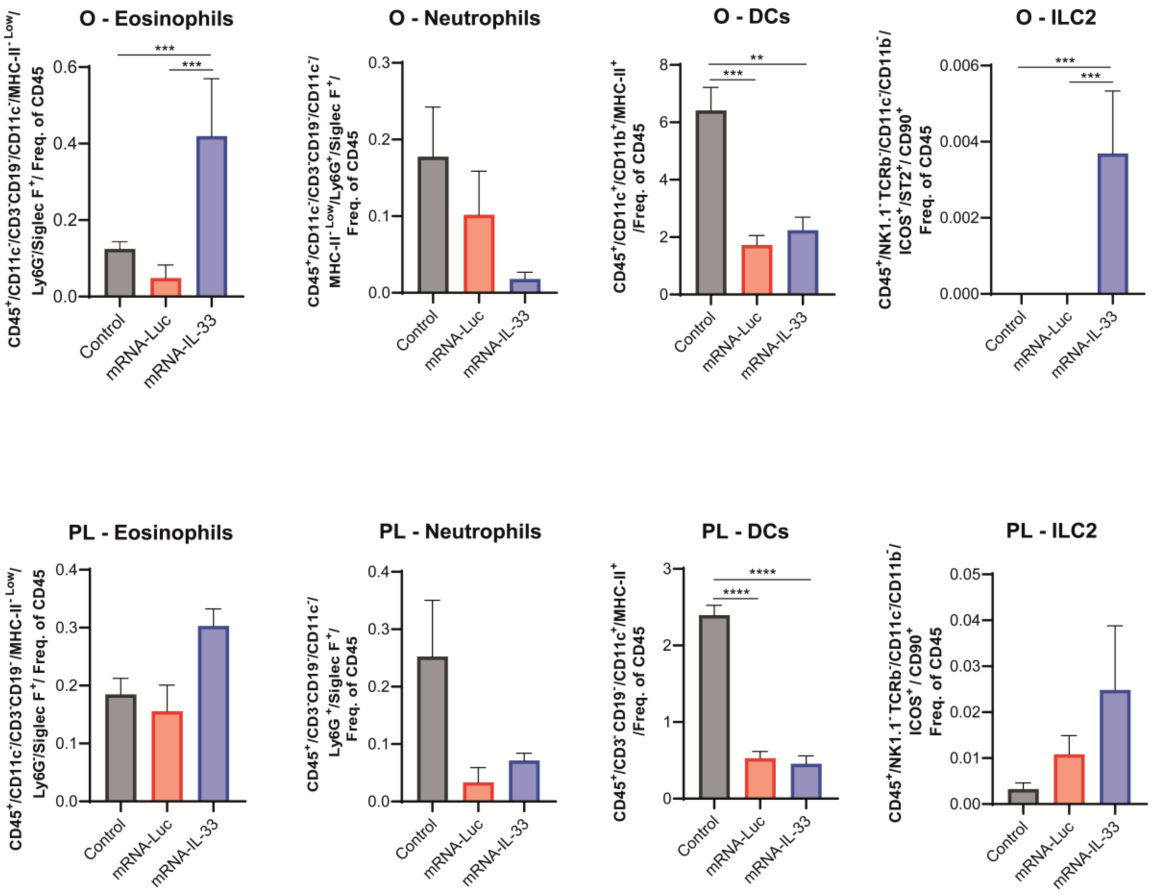

Supplementary Figure 2. Manual flow cytometry analysis of immune populations. C57BL/6 mice (n =6 per group) were injected i.p. with 5×10^5 Panc02.OVA tumor cells. On days 6 and 9 after tumor inoculation, mice received 2.5×10^6 electroporated OT.I T cells. Six days after the second treatment (day 15 post-tumor challenge), mice were sacrificed, and peritoneal lavage fluid and omentum samples were collected for immune profiling by flow cytometry. Conventional gating and flow plots were generated to validate and complement the t-SNE analysis shown in the main figures. Statistical significance was determined via appropriate tests in panel C and F (two-way ANOVA). **p<0.01; ***p<0.001; ****p<0.0001.

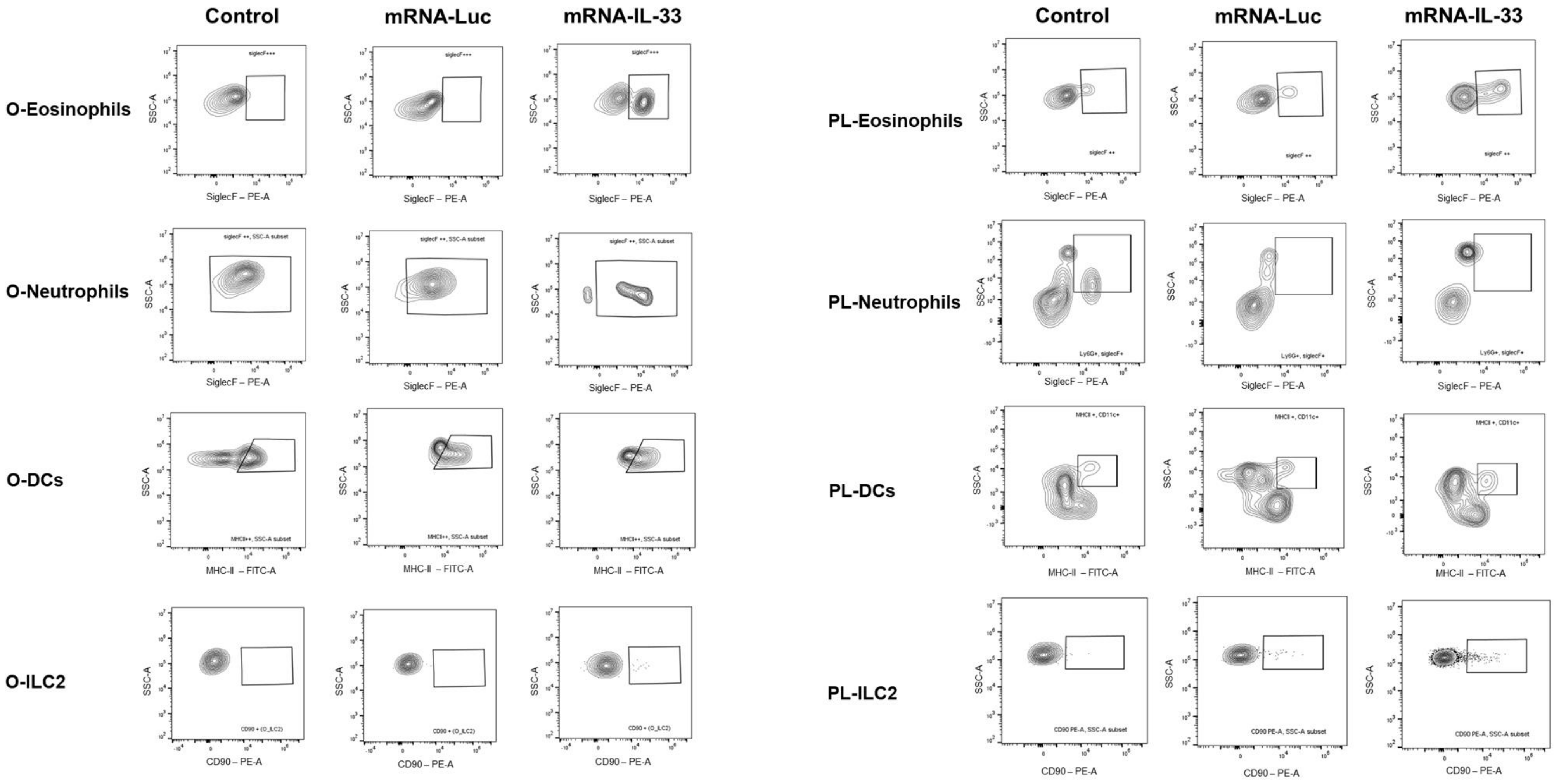

Supplementary Figure 3. Representative dot plots from manual flow cytometry analysis in Supplementary Figure 2.

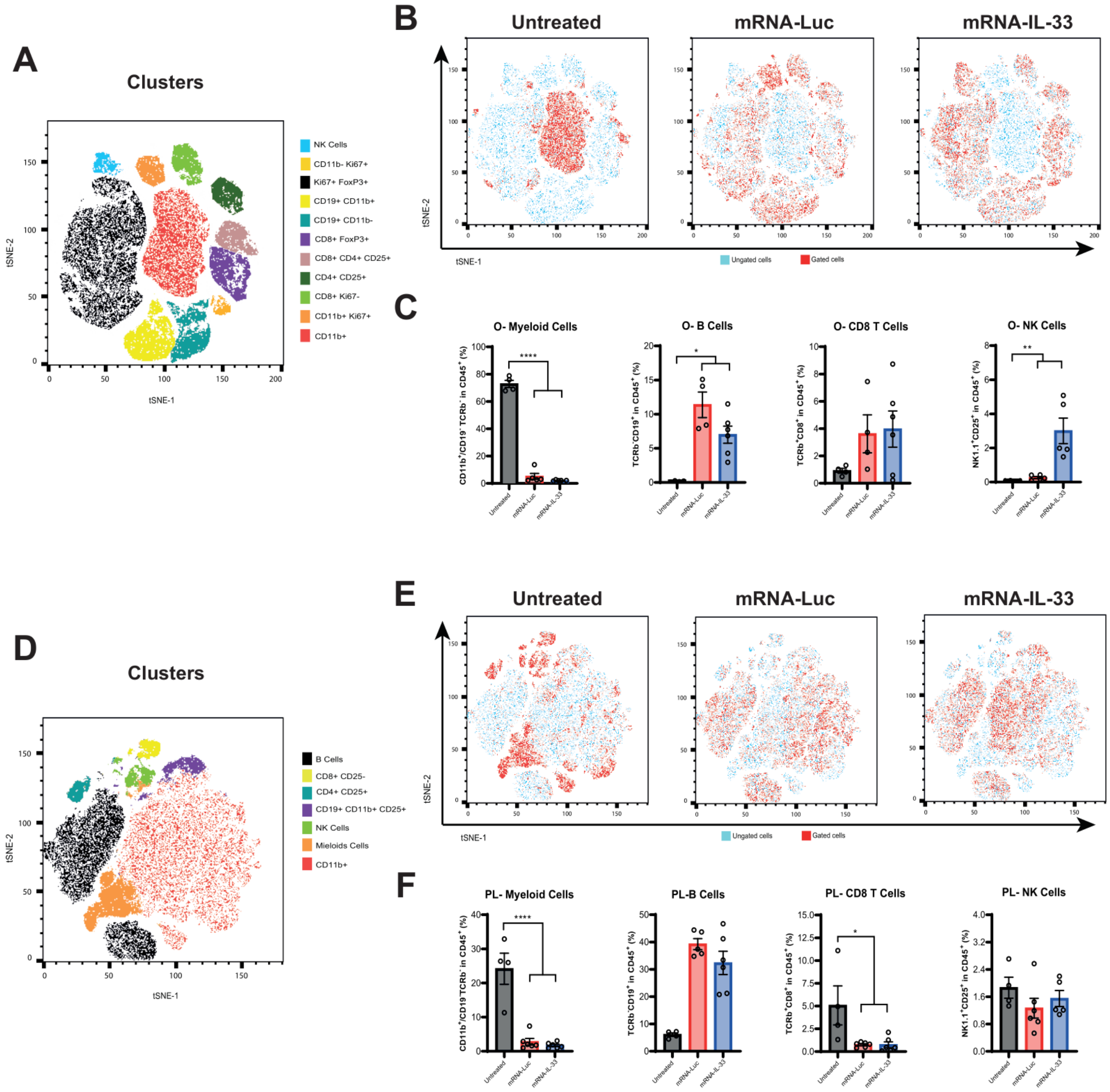

Supplementary Figure 4. Intraperitoneal delivery of IL-33 mRNA via adoptive transfer modulates the tumor microenvironment. C57BL/6 mice (n =6 per group) were injected i.p. with 5 × 10^5 Panc02.OVA tumor cells. On days 6 and 9 post-tumor inoculation, the mice received 2.5 × 10^6 electroporated OT.I T cells. Six days after the second treatment (day 15 after tumor challenge), the mice were sacrificed, and peritoneal lavage samples and omentum were collected for immune profiling via flow cytometry. (**A-B**) t-SNE plots illustrating

immune cell distribution in the omentum (O). (**C**) Percentages of the different immune cell populations in the omentum are shown. (**D-E**) t-SNE analysis was used to evaluate differences in the immune cell populations in peritoneal lavage samples (PL). (**F**) Percentages of the different immune cell populations in the peritoneal lavage samples are displayed. Statistical significance was determined via appropriate tests in panel **C** and **F** (two-way ANOVA). *$p<0.05$; **$p<0.01$; ****$p<0.0001$.

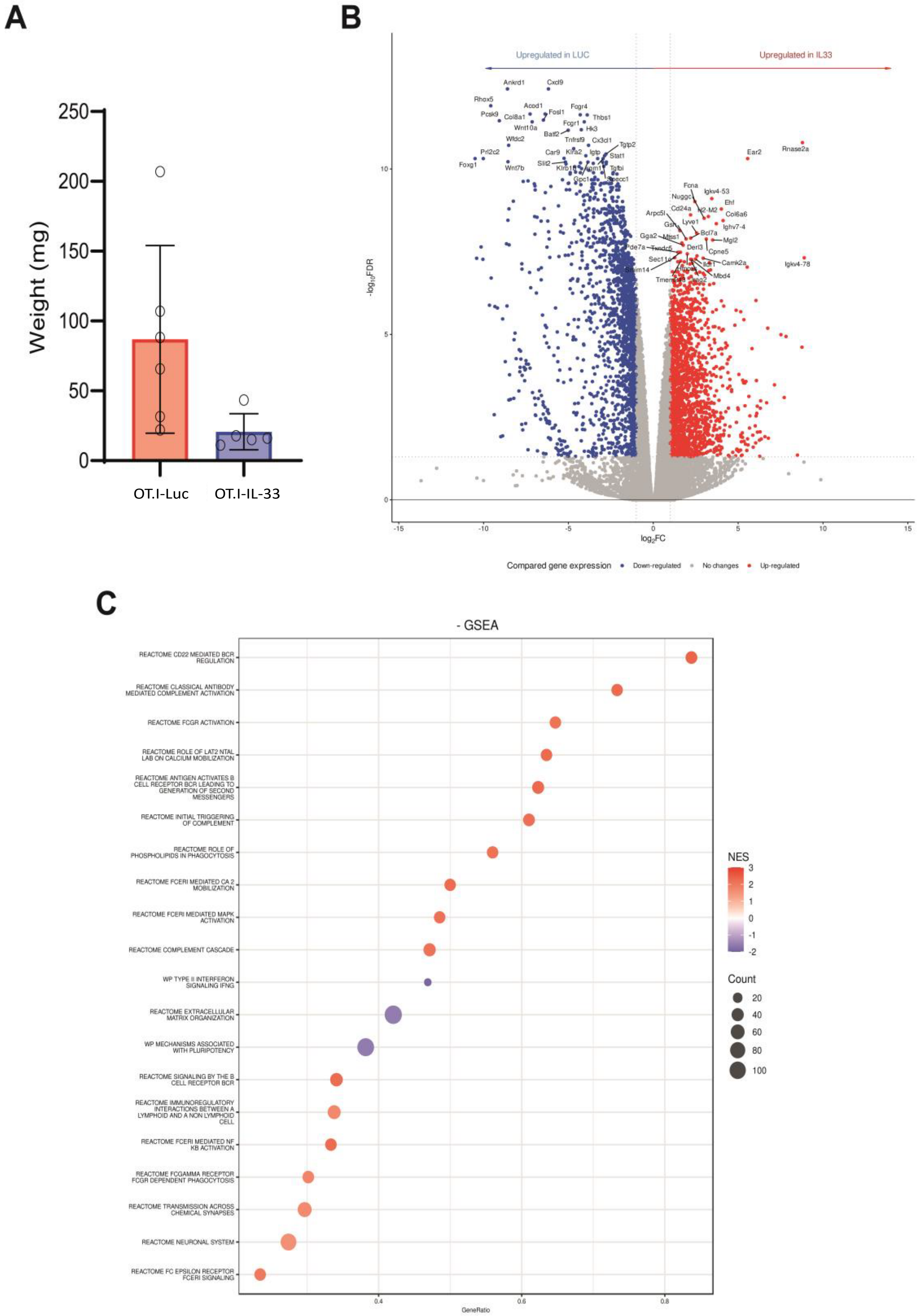

Supplementary Figure 5. RNA-seq analysis of gene expression changes in the omental tumor microenvironment after the treatment with OT.I T cells electroporated with

mRNA-Luc or mRNA-IL-33. C57BL/6 mice (n =4 per group) were injected i.p. with 5 × 10^5 Panc02.OVA tumor cells. On day 6 after tumor inoculation, mice received 2.5 × 10^6 OT.I T cells were electroporated with either Luc mRNA or IL-33 mRNA. Six days after ACT administration (day 15 post-tumor challenge), mice were sacrificed, and the omentum was collected for transcriptomic profiling by RNA sequencing (RNA-seq). (**A**) Omentum weight 14 days after Panc02.OVA tumor challenge. (**B**) Volcano plot showing differentially expressed genes between the mRNA-Luc and mRNA-IL-33 OT.I groups. Genes with a false discovery rate (FDR) < 0.05 are highlighted in red (upregulated) and blue (downregulated). (**C**) Gene Set Enrichment Analysis (GSEA) illustrating the top 20 significantly upregulated and downregulated canonical pathway terms (WikiPathways, Reactome and BioCarta collections from MSigDB) (adjusted p value < 0.05).

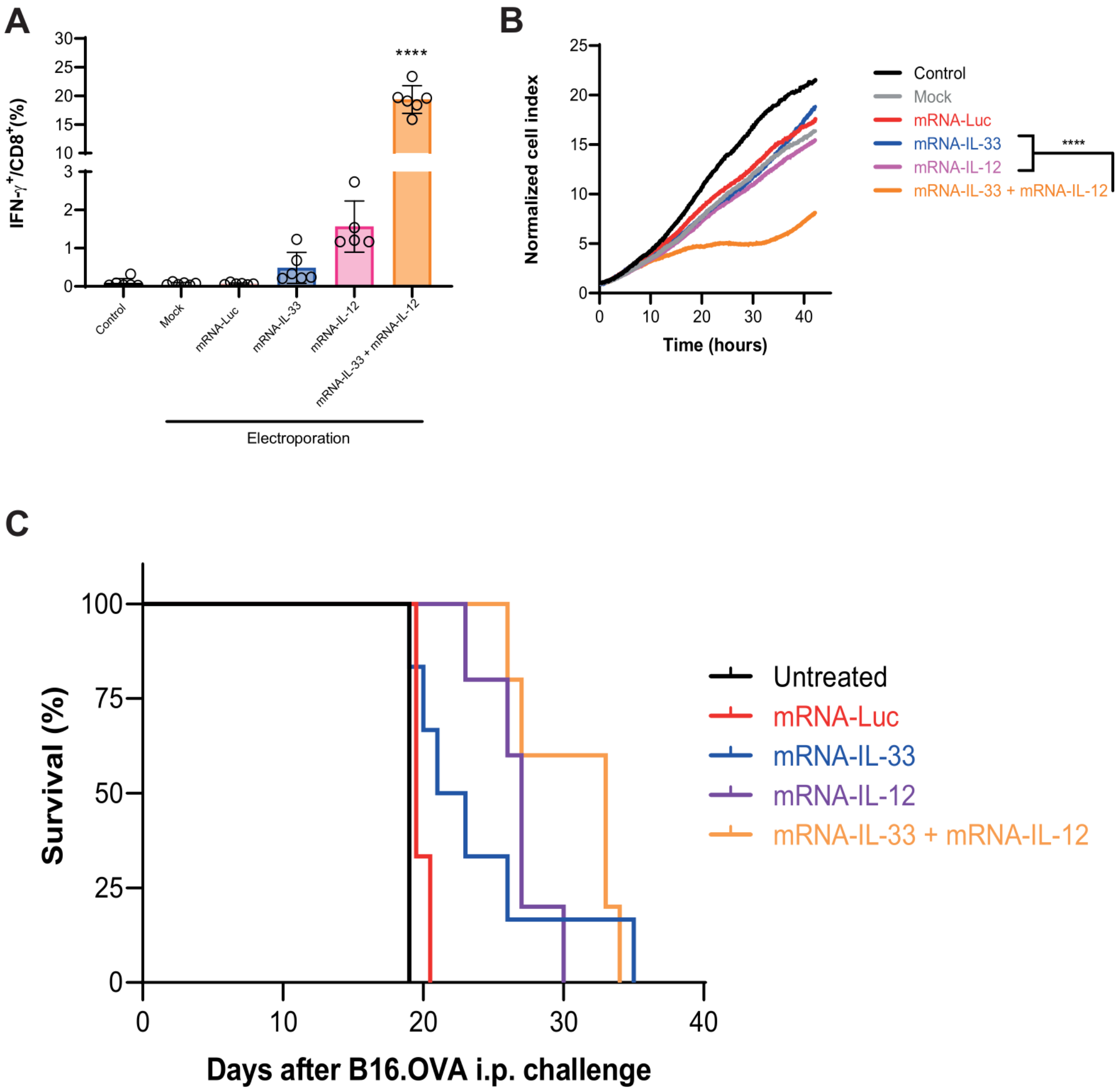

Supplementary Figure 6. Survival analysis of B16.OVA tumor-bearing mice treated with electroporated PMEL-1 T cells. (**A**) Intracellular IFN-γ staining of PMEL-1 T cells 24 h post-electroporation with the indicated mRNAs, analyzed by flow cytometry. (**B**) Cytotoxicity assay using xCELLigence real-time cell analysis. Electroporated PMEL-1 T cells expressing the indicated mRNAs were cocultured with B16.OVA cells at a 1:5 effector-to-target ratio. (**C**) C57BL/6 mice (n = 6 per group) were injected i.p. with 5×10^5 B16.OVA tumor cells. On days 6 and 9, the mice received 2.5×10^6 PMEL-1 T cells i.p., and survival

was monitored over time. Statistical analysis: One-way ANOVA followed by Tukey's multiple comparisons test was applied to panel **A** and **B**. Survival data were analyzed via the log-rank (Mantel-Cox) test (panel **C**). ****$p<0.0001$.
